# Absence of Bim sensitizes mice to experimental *Trypanosoma cruzi* infection

**DOI:** 10.1038/s41419-021-03964-6

**Published:** 2021-07-10

**Authors:** Marcela Hernández-Torres, Rogério Silva do Nascimento, Monica Cardozo Rebouças, Alexandra Cassado, Kely Catarine Matteucci, Maria Regina D’Império-Lima, José Ronnie C. Vasconcelos, Karina R. Bortoluci, José Maria Alvarez, Gustavo P. Amarante-Mendes

**Affiliations:** 1 Instituto de Ciências Biomédicas, Universidade de São Paulo, São Paulo, SP Brazil; 2 Instituto de Investigação em Imunologia, Instituto Nacional de Ciência e Tecnologia (INCT-iii), São Paulo, Brazil; 3 Centro de Terapia Celular e Molecular – CTCMol – Universidade Federal de São Paulo, São Paulo, SP Brazil; 4 Departamento de Farmacologia, Escola Paulista de Medicina, Universidade Federal de São Paulo, São Paulo, Brazil

**Keywords:** Cell death and immune response, Infectious diseases

## Abstract

Chagas disease is a life-threatening disorder caused by the protozoan parasite *Trypanosoma cruzi*. Parasite-specific antibodies, CD8^+^ T cells, as well as IFN-γ and nitric oxide (NO) are key elements of the adaptive and innate immunity against the extracellular and intracellular forms of the parasite. Bim is a potent pro-apoptotic member of the Bcl-2 family implicated in different aspects of the immune regulation, such as negative selection of self-reactive thymocytes and elimination of antigen-specific T cells at the end of an immune response. Interestingly, the role of Bim during infections remains largely unidentified. To explore the role of Bim in Chagas disease, we infected WT, *Bim*^*+/−*^, *Bim*^*−/−*^ mice with trypomastigotes forms of the Y strain of *T. cruzi*. Strikingly, our data revealed that *Bim*^*−/−*^ mice exhibit a delay in the development of parasitemia followed by a deficiency in the control of parasite load in the bloodstream and a decreased survival compared to WT and *Bim*^*+/−*^ mice. At the peak of parasitemia, peritoneal macrophages of *Bim*^*−/−*^ mice exhibit decreased NO production, which correlated with a decrease in the pro-inflammatory Small Peritoneal Macrophage (SPM) subset. A similar reduction in NO secretion, as well as in the pro-inflammatory cytokines IFN-γ and IL-6, was also observed in *Bim*^*−/−*^ splenocytes. Moreover, an impaired anti-*T. cruzi* CD8^+^ T-cell response was found in *Bim*^*−/−*^ mice at this time point. Taken together, our results suggest that these alterations may contribute to the establishment of a delayed yet enlarged parasitic load observed at day 9 after infection of *Bim*^*−/−*^ mice and place Bim as an important protein in the control of *T. cruzi* infections.

## Introduction

*Trypanosoma cruzi* is the etiological agent of Chagas disease, a relevant problem of global health. At present, 6–7 million people are infected with the protozoan and more than 65 million are at risk, especially in Latin America where the disease is endemic [1]. Because it has no spontaneous cure, Chagas disease remains one of the most important neglected diseases in the world, as recognized by the World Health Organization [2]. Along its life cycle in the insect vector and in the mammalian host, *T. cruzi* develops complex morphological changes that allow its replication and survival in different environmental conditions. In mammals, these forms include the intracellular amastigotes—a replicative form of the parasite, and the extracellular trypomastigotes present in the blood and tissues [3].

Control of *T. cruzi* parasites relies on both innate and adaptive immune responses [4]. Macrophages, both CD4^+^ and CD8^+^ T cells, as well as B cells, contribute to parasite destruction [5–7]. IFN-γ, mainly produced by activated CD4^+^ and CD8^+^ T cells, increases the effector activity of macrophages, leading to the production of trypanocidal molecules such as nitric oxide [8, 9]. Also, cytotoxic CD8^+^ T cells contribute to *T. cruzi* control through the recognition and destruction of cells that harbor intracellular forms of the parasite [9]. Interestingly, one of the mechanisms *T. cruzi* employs to avoid immune response is the induction of apoptosis in B and T cells, which has important implications to the pathogenesis of Chagas disease [10–12].

Bim is a potent pro-apoptotic molecule of the Bcl-2 family that participates in the induction of intrinsic/mitochondrial pathway of apoptosis. In vitro, Bim is essential for the induction of apoptosis in different cell types, including lymphocytes, mast cells, epithelial cells, endothelial cells, macrophages, granulocytes, and neurons [13]. In vivo, Bim is a critical regulator of the immune response as it participates at the negative selection of self-reactive thymocytes, activated T-cell autonomous death, regulation of T memory cells, and deletion of autoreactive B cells, among other functions [14, 15]. The number of granulocytes, monocytes, T lymphocytes (CD4^+^ and CD8^+^), and B lymphocytes increase two to fourfold in *Bim*^*−/−*^ mice [16]. With age, mice develop splenomegaly, lymphadenopathy, hypergammaglobulinemia, and a systemic autoimmune disease similar to Systemic Lupus Erythematosus in humans [17].

Regarding the control of infections, the current view is that Bim limits protective immunity. Indeed, in mice infected with *Leishmania major*, the absence of this pro-apoptotic protein resulted in increased frequency of antigen-specific CD4^+^ T cells at later stages of infection and consequent increased resistance to this parasite [18]. Likewise, Bim was shown to be a critical factor that renders macrophages susceptible to virulent strains of *Mycobacterium tuberculosis* thereby promoting apoptosis of infected cells and consequent spread of the bacteria [19]. In contrast to these studies, we found that Bim is a rather positive regulator of immunity to *T. cruzi*. *Bim*^*−/−*^ mice are less efficient in controlling blood parasites, which significantly impact on overall survival. Macrophages obtained from the peritoneal cavity of *Bim*^*−/−*^ mice at the peak of parasitemia displayed impaired NO production. Moreover, splenocytes from *Bim*^*−/−*^ mice showed reduced NO production, in addition to lower levels of IFN-γ and IL-6 secretion. Finally, we also found a compromised anti-*T. cruzi* CD8^+^ T-cell response in *Bim*^*−/−*^ mice. Taken together, our study extends the knowledge of the role of Bim on the immune response to infectious diseases and suggests a relevant function of this molecule in the control of *T. cruzi* experimental infection.

## Materials and methods

### Mice

Mice were bred in our animal facilities and maintained under specific-pathogen-free conditions. *Bim*^*−/−*^ mice were kindly provided by Dr. Philippe Bouillet (The Walter and Eliza Hall Institute of Medical Research, Melbourne, Australia). Due to an impaired embryonic development of the vagina of most *Bim*^*−/−*^ female mice, *Bim*^*−/−*^ males and *Bim*^*+/−*^ females were crossed in order to obtain the largest possible number of offspring. Both genotypes were identified by conventional PCR using tail DNA. Age-matched female mice were used between 8 and 10 weeks old to avoid the inherent splenomegaly, lymphadenopathy, and hypergammaglobulinemia that progressed with age in these mice [17].

### Parasites and infection

Mice were infected intraperitoneally (i.p.) with 1 × 10^4^ trypomastigotes of the Y strain of *T. cruzi* obtained from the blood of A/J mice previously infected for 7 days. Parasitemia and mortality were monitored daily and individually until parasites were undetectable in the blood. For that, blood smear slides were made with 5 µL of blood obtained from the tail vein of each animal, and a parasitemia was provided by counting the number of mobile parasites in fifty random fields under a ×40 objective.

### Nitric oxide measurement

Levels of NO were indirectly determined by the quantification of nitrite in the culture supernatant of peritoneal exudate cells (PECs) or splenocytes by the Griess reaction [20].

### Phenotyping of peritoneal macrophages

PECs were obtained from peritoneal lavage with 5 mL of cold sterile phosphate-buffered saline (PBS), washed and resuspended in RPMI-1640 medium supplemented with 10% fetal calf serum (FCS). Cells were stained with fluorescence-labeled monoclonal antibodies according to Table 1.

**Table 1:** Antibodies used to immunophenotyping peritoneal macrophages.

Antibody	Clone	Flourochrome	Supplier
CD19	1D3	PECy7	BD Bioscience
CD11c	HL3	APC	BD Bioscience
F4/80	6F12	PE	BD Bioscience
MHC-II	M5/114.15.2	FITC	BioLegend

Samples were analyzed by flow cytometry using a FACSCanto II (Becton Dickinson) device and the FlowJo (TreeStar) software. Gating strategy used was FSC-A x FSC-H to exclude doublets, CD19 x FSC-H to eliminate B lymphocytes, CD11c x FSC-A to exclude dendritic cells, F4/80 x SSC-A to select the population of F4/80^+^ macrophage, and F4/80 x MHC-II to determine the subpopulation of peritoneal macrophages: SPM (small peritoneal macrophage) and LPM (large peritoneal macrophage) (20,21).

### Determination of cytokine production

Splenocytes from C57BL/6 WT, *Bim*^*+/−*^, and *Bim*^*−/−*^ mice previously (9 days before) inoculated or not with *T. cruzi* were cultured for 24 h (IL-6) and 48 h (IFN-γ), and supernatants were analyzed by capture ELISA using the commercial kits from BD Bioscience (OptEIA™).

### In vivo cytotoxicity assay

Effector activity of antigen-specific CD8^+^ T lymphocytes was evaluated by the in vivo cytotoxicity assay, as previously described [21]. Briefly, WT, *Bim*^+/−^, and *Bim*^*−/−*^ recipient mice were infected or not with *T. cruzi*. Target cells were prepared using splenocytes from WT mice labeled with 1 μM CFSE Cell Proliferation Kit (Molecular Probes) or 1 or 10 μM CellTrace™ Violet Cell Proliferation Kit (Molecular Probes) (CTV^low^ or CTV^high^, respectively) in PBS for 15 min at 37 °C. After centrifugation at 500 × *g* for 5 min, the CTV^high^ and CTV^low^ populations were resuspended in RPMI-10% FCS and pulsed for 1 h at 37 °C with 0.0001 μM or 0.01 μM of the *T. cruzi* PA8 H-2K^b^ (VNHRFTLV) immunodominant peptide. The CFSE^low^ cells remained unpulsed and served as a control, non-target population. After this step, the pulsed cells were washed three times, centrifuged at 500 × *g* for 5 min, and the three tubes were mixed at the same cell proportion (1:1:1). Finally, each recipient mouse received 3 × 10^7^ cells in 200 μL of RPMI-1640 by retro-orbital route. After 16–20 h, splenocytes from recipient mice were collected, processed, washed with RPMI-10% FCS, and fixed with 1% paraformaldehyde solution in PBS. Samples were analyzed by flow cytometry on a FACSCanto II (Becton Dickinson) and subsequently in the FlowJo software (TreeStar). The gating strategy used was FSC-A x FSC-H to exclude doublets, FSC-A x SSC-A to exclude debris, and finally CFSE x CellTrace to determine the splenocytes populations stained with the vital dyes. Ten thousand events were acquired on the CFSE^low^ population that represents the non-target cells. The percentage of specific lysis was determined using the following formula:

$${\mathrm{\% }}\,{\mathrm{Specific}}\,{\mathrm{lysis}} = \left[ {1 - \frac{{{\mathrm{\% }}{\mathrm{CTV}}_{{\mathrm{high}}\,{\mathrm{or}}\,{\mathrm{low}}}\,{\mathrm{infected}}/{\mathrm{\% }}{\mathrm{CFSE}}_{{\mathrm{low}}}\,{\mathrm{infected}}}}{{{\mathrm{\% }}{\mathrm{CTV}}_{{\mathrm{high}}\,{\mathrm{or}}\,{\mathrm{low}}}\,{\mathrm{naive}}/{\mathrm{\% }}{\mathrm{CFSE}}_{{\mathrm{low}}}\,{\mathrm{naive}}}}} \right]\times100$$

### Enzyme-linked immunospot (ELISPOT) assay

The ELISPOT assay was performed in order to determine the frequency of PA8-specific IFN-γ producing cells based on the protocol previously described [22]. Responder cells (1 × 10^6^ cells/mL) were obtained from the spleens of WT, *Bim*^*+/−*^ and *Bim*^*−/−*^ mice previously infected or not with 1 × 10^4^ trypomastigotes of the Y strain of *T. cruzi*. Splenocytes of C57BL/6 mice were used as antigen-presenting cells (3 × 10^6^ cells/mL). One hundred microliters of each cellular suspension (responder and antigen-presenting cells) were incubated or not with PA8 peptide in a final concentration of 10 μM for 24 h at 37 °C and 5% CO_2_ in static conditions.

### Statistical analysis

Statistical analysis was performed using two-way ANOVA and Bonferroni´s post-test. The Log-Rank test was used to compare mouse survival between groups. All analysis was performed with the software Graphpad Prism, version 5. The difference was considered significant when the *p* value was <0.05.

## Results

### *Bim*^*−/−*^ mice are susceptible to *T. cruzi* infection

**Fig. 1:**
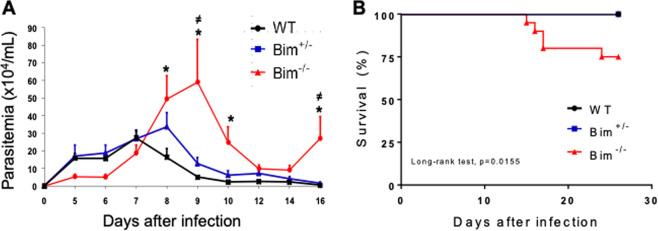
*Bim*^*−/−*^ mice are susceptible to *T. cruzi* infection. C57BL/6 WT, *Bim*^*+/−*^, and *Bim*^*−/−*^ mice were inoculated i.p. with 1 × 10^4^ trypomastigotes of the Y strain of *T. cruzi*. **A** Parasitemia was monitored daily from day 5 to 16 after infection. The results are expressed as the mean and standard deviation for each group (*n* = 10) and are representative of four independent experiments. The asterisk (*) represents statistically significant differences between *Bim*^*−/−*^ and WT mice and the ≠ symbol indicates statistically significant differences between *Bim*^*−/−*^ and *Bim*^*+/−*^ groups. Statistical analysis was performed using two-way ANOVA, followed by Bonferroni post-test (*p* < 0.05). **B** Survival was monitored daily for 26 days. The results represent sum of three different experiments (*n* = 20 for *Bim*^*+/+*^ and *Bim*^*−/−*^ groups and *n* = 10 for *Bim*^*+/−*^ mice). Kaplan–Meier curve (log-rank test).

To investigate the role of Bim during *T. cruzi* infection, we first analyzed the kinetics of parasitemia in WT*, Bim*^*+/−*^, and *Bim*^*−/−*^ mice inoculated as described above. WT mice showed a peak parasitemia on day 7 p.i. followed by a reduction of the number of parasites in the bloodstream from day 8 to day 16 after infection (Fig. 1A). We observed no statistically significant difference between WT and *Bim*^+/−^ mice (Fig. 1A). Interestingly, *Bim*^*−/−*^ mice showed a delayed control of parasitemia reaching a later and higher peak around day 9 p.i. (Fig. 1A). In addition, *Bim*^*−/−*^ mice showed a deficiency to regulate parasitemia and around 25% of animals failed to control the infection, showing a second peak of parasitemia and dying from day 15 after the initial challenge (Fig. 1B). The remaining 75% controlled the number of parasites to the point of becoming undetectable and survived throughout the course of the disease (data not shown). Our data suggest that Bim is important for the control of parasitemia and its absence impairs survival during the acute phase of *T. cruzi* infection.

### *Bim*^*−/−*^ PECs displayed impaired NO secretion associated with reduced percentage of small peritoneal macrophages (SPM)

**Fig. 2:**
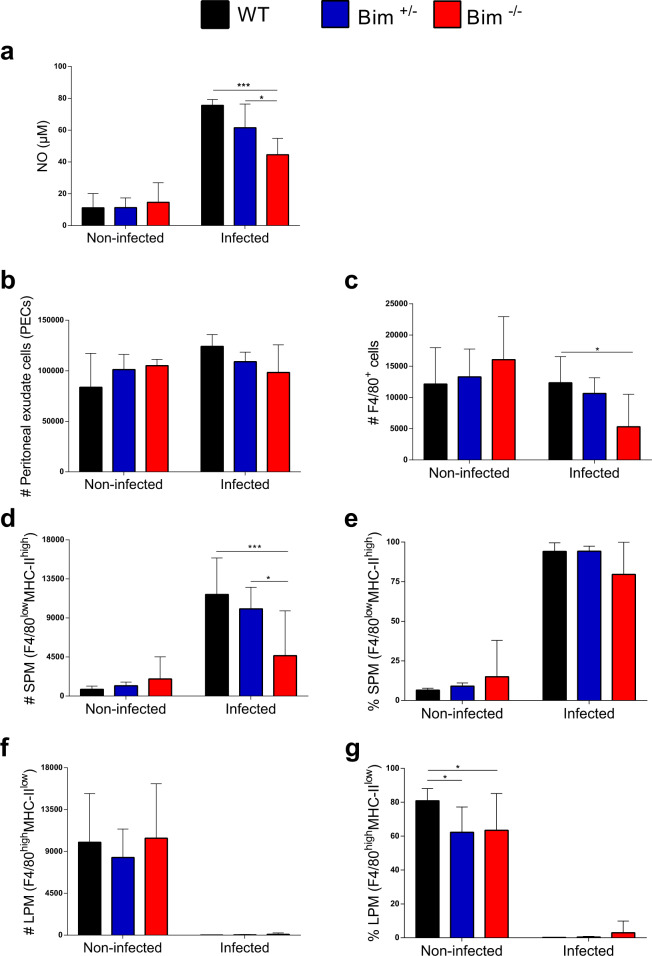
Impaired NO secretion and deficient influx of SPM subpopulation to the peritoneal cavity of *Bim*^*−/−*^ mice at peak parasitemia. WT, *Bim*^*+/−*^, and *Bim*^*−/−*^ C57BL/6 mice were infected (i.p.) or not with 1 × 10^4^ trypomastigotes of the Y strain of *T. cruzi*. After 9 days, PECs were isolated, cultured for 48 h with no further stimulation, and the concentration of nitric oxide was determined by the Griess reaction (**A**). In addition, PECs were labeled with a mixture of monoclonal antibodies against CD19, CD11c, F4/80, and MHC-II and analyzed by flow cytometry. The gating strategy used was based on FSC-A x FSC-H to exclude doublets, CD19 x FSC-H to exclude B cells, CD11c x FSC-A to exclude dendritic cells, F4/80 x SSC-A to select the population of F4/80^+^ macrophages and, finally, F4/80 x MHC-II to analyze the percentage and distribution of SPM and LPM subpopulations (**B**–**G**). The results are expressed as the average and standard deviation of 5 animals for the infected groups and 3 animals for the non-infected groups. All results are representative of 3 independent experiments and are expressed as the mean and standard deviation of triplicate samples. Statistical analysis was performed using two-way ANOVA, followed by Bonferroni post-test. *p < 0.05, ***p < 0.001.

Because *T. cruzi* is inoculated i.p. in our experimental system we decided to investigate the activation status of peritoneal macrophages on day 9 of infection, which correspond to the peak parasitemia of *Bim*^*−/−*^ mice. PECs from *Bim*^*−/−*^ mice showed a significant decrease in NO production compared to WT and *Bim*^*+/−*^ counterparts (Fig. 2A), which could explain the deficient control of infection in *Bim*^*−/−*^ mice.

F4/80^+^ peritoneal macrophages are composed by the Large Peritoneal Macrophage (LPM) and the Small Peritoneal Macrophage (SPM), which differ in size, in phenotypic markers and in their functionality [23]. To characterize the macrophage subsets in the peritoneal cavity of our experimental groups, PECs from WT, *Bim*^*+/−*^, and *Bim*^*−/−*^ mice infected or not 9 days before were stained for CD19, CD11c, F4/80, and MHC-II and analyzed by flow cytometry. First, we observed no difference in the number of PECs in all condition tested (Fig. 2B). Interestingly, the number of F4/80^+^ cells was lower in infected *Bim*^*−/−*^ peritoneal cavity (Fig. 2C). The SPM population was identified by the expression of F4/80^low^ and MHC-II^high^ and the LPM population by the expression of F4/80^high^ and MHC-II^low^ as previously described [23–25]. As expected, all non-infected groups showed a predominance of LPMs over the SPM population (Fig. 2D–G). In addition, there were no significant differences between non-infected WT, *Bim*^*+/−*^, and *Bim*^*−/−*^ mice, suggesting that Bim does not interfere with homeostatic distribution of macrophage subpopulations in the peritoneal cavity (Fig. 2D–G). Moreover, as we previously demonstrated [23], the distribution of macrophage subpopulations was greatly modified by infection, with predominance of the SPM (the inflammatory-prone subset) and virtual absence of LPM (Fig. 2D–G). Notably, *Bim*^*−/−*^ infected mice showed a significantly reduced percentage and total number of SPM population compared to the other groups (Fig. 2D–G). These results suggest that *Bim*^*−/−*^ infected mice displayed an impaired influx of SPM to the peritoneal cavity in response to *T. cruzi* infection. Lower numbers of the inflammatory SPMs could explain the reduced production of NO observed in *Bim*^*−/−*^ PECs at the peak of parasitemia (Fig. 2A) and contribute to the deficient control of *T. cruzi* observed in these mice.

### Splenocytes of *Bim*^*−/−*^ mice have a deficiency in the production of NO, IFN-γ, and IL-6 in the acute phase of *T. cruzi* infection

**Fig. 3:**
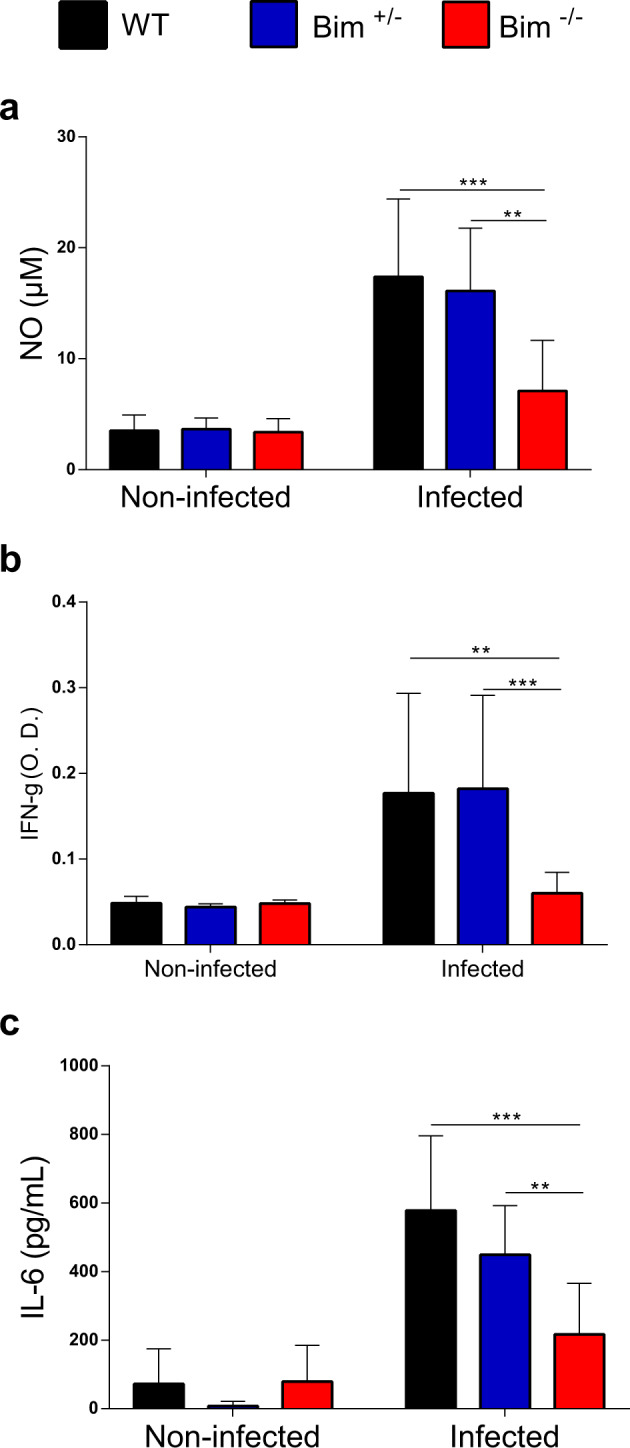
Splenocytes from *Bim*^*−/−*^ mice are deficient in NO, IFN-γ, and IL-6 production at peak parasitemia. Supernatants of splenocyte cultures of WT, *Bim*^*+/−*^, and *Bim*^*−/−*^ C57BL/6 mice previously inoculated or not (9 days before) with 1 × 10^4^ trypomastigotes of the Y strain of *T. cruzi* were collected after 24 h (IL-6) and 48 (NO, IFN-γ) of incubation. **A** The concentration of nitric oxide (NO) was determined by the Griess reaction. **B**, **C** IFN-γ and IL-6 concentration were determined by capture ELISA assay. Statistical analysis was performed using two-way ANOVA, followed by Bonferroni post-test. *p < 0.05, ***p < 0.001.

To investigate whether the deficient response observed in *T. cruzi*-infected *Bim*^*−/−*^ mice was restricted to the peritoneal cavity or disseminated to other immunological tissues/organs, we evaluated NO, IFN-γ, and IL-6 secretion by splenocytes. After 9 days of infection, we isolated splenocytes from WT, *Bim*^*+/−*^, and *Bim*^*−/−*^ C57BL/6 mice. Forty-eight hours after in vitro culture, the concentration of NO was determined by the Griess reaction. Again, we observed a statistically significant decrease in the production of NO by *Bim*^*−/−*^ splenocytes, compared to the control groups (Fig. 3A). As shown in Fig. 3B, C, splenocytes from *Bim*^*−/−*^ mice also showed impaired release of IFN-γ and IL-6. These results suggest that the deficiency in effector function of *Bim*^*−/−*^ immune cells after 9 days of infection with *T. cruzi* were not restricted to the peritoneal cavity.

### *Bim*^*−/−*^ mice have impaired antigen-specific CD8^+^ T cells response against *T. cruzi*

**Fig. 4:**
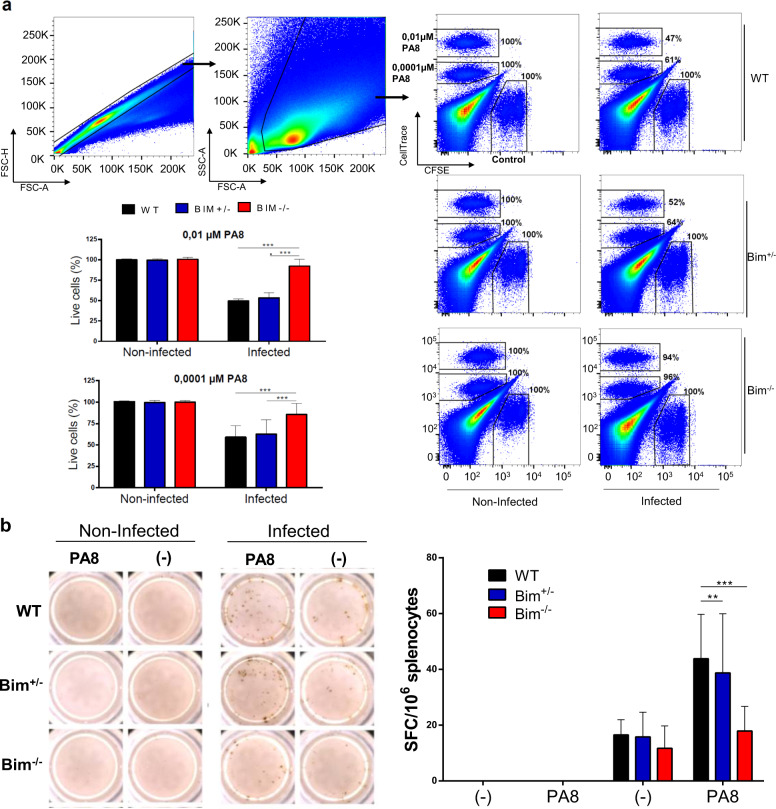
*Bim*^*−/−*^ mice have impaired antigen-specific CD8^+^ T cells response against *T. cruzi* in the acute phase of infection. C57BL/6 WT*, Bim*^*+/−*^, and *Bim*^*−/−*^ mice were inoculated i.p. with 1 × 10^4^ trypomastigotes of the Y strain of *T. cruzi*. **A** Eight days after infection, mice were inoculated with 3 × 10^7^ splenocytes divided into three populations (1:1:1) stained with 1 μM of CFSE (control), 1 μM of CellTrace violet (CTV^low^ - pulsed with 0.0001 μM of PA8 peptide) and 10 μM of Cell trace (CTV^hi^ - pulsed with 0.01 μM of PA8 peptide). Sixteen to twenty hours after cell transfer splenocytes were harvested and specific lysis of target cells was determined by flow cytometry. **B** The frequency of IFN-γ producing cells was measured ex vivo in the presence of PA8 peptide by ELISPOT assay. SFC (Spot forming cells). The results are expressed as the media and standard deviation of five animals for the infected group and three animals for the non-infected group. The results are representative of three independent experiments. Statistical analysis was performed using two-way ANOVA, followed by Bonferroni post-test. **p < 0.01, ***p < 0.001.

Finally, it is well established that antigen-specific CD8^+^ T cells are essential for the control of *T. cruzi* infection. Because splenocytes from *T. cruzi*-infected *Bim*^*−/−*^ mice displayed an impaired IFN-γ secretion, we investigated the in vivo *T. cruzi*-specific CD8^+^ T-cell cytotoxic response in these mice. To look at the ability of CD8 T cells to in vivo kill targets presenting antigens from *T. cruzi*, we performed in vivo cytotoxicity assays at the peak of parasitemia, i.e., nine days after infection. Our results showed that *Bim*^*−/−*^ mice have reduced capacity to eliminate target cells (CellTrace^high^ and CellTrace^low^) pulsed with the PA8 peptide from ASP2, a major antigen of Y strain *T. cruzi* recognized by the CD8 T-cell compartment of C57BL/6 mice [26] (Fig. 4A). In parallel, we assessed the frequency of PA8-specific IFN-γ-producing cells by ELISPOT assay. As shown in Fig. 4B, in the absence of in vitro restimulation, all infected animals have a similar low/undetectable frequency of IFN-γ-producing cells. Importantly, after stimulation with PA8 peptide, the frequency of IFN-γ-producing cells of *Bim*^*−/−*^ mice was significantly lower than that observed in WT e *Bim*^*+/−*^ groups. Taken together, our results suggest that Bim regulates the immune response to *T. cruzi* at multiple levels and its absence renders C57BL/6 mice susceptible to the infection.

## Discussion

Understanding the pathophysiological aspects of Chagas disease, particularly the molecular mechanisms implicated in resistance or susceptibility to infection, will help us designing better pharmacological strategies to fight this chronic disease [27]. In this work, we observed that the absence of Bim, a pro-apoptotic, BH3-only member of the Bcl-2 family, renders mice susceptible to *T. cruzi*. The peak of parasitemia of Bim-deficient mice (day 9) is delayed in 2 days, compared to Bim-proficient mice (day 7). During this time, the parasitemia continues to grow and reaches a significantly higher blood concentration. Most mice finally control the infection to the point where no parasites can be detected in the blood by direct examination at the microscope. However, in around 25% of *Bim*^*−/−*^ mice, parasitemia rises again and mice die at around day 16 post infection. *Bim*^*−/−*^ mice displayed an impaired systemic macrophage and CD8 T-cell effector responses to *T. cruzi* at the peak of parasitemia compared to WT or heterozygous mice. While still elusive, the molecular relationship between the expression of Bim and the in vivo activation of macrophages and CD8 T cells in response to *T. cruzi* is currently under investigation in our laboratory.

Bim is a pro-apoptotic member of the Bcl-2 family. The major role of Bim in the immune system has been ascribed to the control of B and T lymphocyte expansion, as well as thymocyte maturation [14, 15]. With age, *Bim*^*−/−*^ mice develop splenomegaly and lymphadenopathy due to uncontrolled proliferation of B and T cells, monocytes and granulocytes [16]. Interestingly enough, *Bim*^*−/−*^ mice infected with *Leishmania major*, showed improved protection against reinfection with the parasite [18]. In contrast to our results, the authors observed a significant increase in the production of IFN-γ in the lymph nodes, as well as in the number of specific CD4 T lymphocytes producing IFN-γ at the site of infection, along with a decrease in the production of IL-10 [18]. These results are in agreement with data showing that in the absence of Bim there is an increase in the clonal expansion of antigen-specific CD4 T lymphocytes, which contributes to the improved resistance to the parasite after a second infection. At a glance, our data may appear to conflict with these observations. However, it is important to note that we evaluated the primary response against *T. cruzi*, whereas the work by Reckling et al. [18] assessed the secondary response to *L. major*. In this regard, it would be interesting to compare the response of recovered WT, *Bim*^*+/−*^ and *Bim*^*−/−*^ mice to a second, perhaps lethal infection with *T. cruzi*. It is reasonable to imagine that under these circumstances *Bim*^*−/−*^ mice that recovered from the first infection with *T. cruzi* would develop a larger immune response to a secondary challenge with the same parasite, similarly to the work by Reckling et al. with *L. major* [18].

Peritoneal macrophages are the first cells to respond to the experimental infection with *T. cruzi*, as parasites are inoculated i.p., and their inflammatory activation exerts microbicidal effects largely through the production of NO [28]. Mice treated with NO synthase enzyme inhibitors have been shown to be highly susceptible to increased parasitemia and mortality after infection with the Y strain of *T. cruzi* [29]. In our study, we observed a reduced NO secretion from *Bim*^*−/−*^ PECs at the peak of parasitemia, which seems to reflect a deficient peritoneal macrophage activation in response to *T. cruzi* in vivo and could explain the high levels of parasites in the blood of *Bim*^*−/−*^ mice. We considered the possibility that the in vivo reduced NO production was related to differences in the dynamic/composition of macrophage subpopulations in these animals. In this regard, there are two subtypes of peritoneal macrophages that exhibit distinct morphologies, phenotypes, and biological activities: SPM and LPM. Importantly, their population dynamics changes with different inflammatory or infectious stimulus [23, 24]. Under physiological conditions, LPM represents the most abundant population whereas upon inflammatory stimuli or in response to infections SPM become the predominant subtype and the major source of inflammatory mediators [23]. Particularly, it has been shown that mice infected with *T. cruzi* have elevated numbers of SPM that secrete high levels of NO, mainly in response to LPS and IFN-γ [23]. Notably, SPM are inflammatory macrophages able to capture and process antigens in vivo [30] and was shown to be involved in the activation of CD4 T lymphocytes, which in turn improve the humoral immune response [24]. Interestingly, we observed that *Bim*^*−/−*^ mice have a reduced percentage of the SPM population when compared to the WT and *Bim*^*+/−*^ counterparts at the peak of parasitemia.

Besides the potential deficient local response at the peritoneal cavity, *Bim*^*−/−*^ splenocytes also showed a reduction in NO production as well as inflammatory cytokines such as IFN-γ and IL-6, compared to control animals. IL-6 is an important pro-inflammatory cytokine that induces the differentiation of CD8^+^ T cells into cytotoxic T cells [31]. Splenocyte deficiency in IL-6 and/or IFN-γ could also contribute to the establishment of the high parasitic load observed 9 days after infection and/or to the deficit to fully control parasitemia observed in 25% of mice that succumb from the disease. Different studies have shown that production of IFN-γ is correlated with resistance and control of the parasite in the acute phase of *T. cruzi* infection [28, 32, 33]. Furthermore, exogenous administration of recombinant IFN-γ to *T. cruzi*-infected mice is associated with macrophage upregulation of iNOS and NOX2 and consequent production of NO and ROS, respectively, resulting in reduced parasitemia and mortality [34].

Besides releasing effector cytokines such as IFN-γ and TNF-α, CD8 T lymphocytes are crucial element of the adaptive immunity against *T. cruzi* by virtue of their cytotoxic activity against infected cells [35, 36]. Our data revealed that at the peak of parasitemia *Bim*^*−/−*^ mice exhibited a reduced capacity to eliminate *T. cruzi*-specific targets in vivo. This could be the result of deficient activation/expansion/differentiation of antigen-specific CD8 T cells or defective cytotoxic machinery, or both. At this point, we have not yet investigated the molecular composition of cytotoxic granules of *Bim*^*−/−*^ CD8 T cells. However, our results clearly showed that the frequency of *T. cruzi*-specific *Bim*^*−/−*^ CD8 T cells, as evaluated by ELISPOT analysis of IFN-γ-producing CD8 T cells, is decreased at the peak of parasitemia, compared to WT and *Bim*^*+/−*^ counterparts.

In contrast to IFN-γ or CD8 T-cell deficiencies, where the mortality rate of mice is virtually 100% [26], Bim elimination resulted in a somewhat mild vulnerability to experimental *T. cruzi* infection. In fact, although *Bim*^*−/−*^ mice are indeed deficient in IFN-γ secretion and CD8 T-cell-mediated cytotoxic activity, both arms of the immune response do exist to a great extent in these animals. This may be the reason why only a fraction of *Bim*^*−/−*^ mice succumbs from the disease, despite all the features of an immunocompromised animal that we unveiled. In any case, around 75% of *Bim*^*−/−*^ mice somehow manage to mount a productive immune response against *T. cruzi* and live at least another 6–8 months without showing any sign of the disease. Still, we do not have the explanation for the 25% of mice that fail to control parasitemia and die shortly afterward. We believe that although genetically identical our *Bim*^*−/−*^ mice display different immunological fitness due to environmental factors such as stress and/or nutritional fortitude or to a slightly different T and B cell repertoire due to stochastic rearrangements of the T and B cell receptors. Our hypothesis is that part of the Bim-deficient mice achieves only a borderline level of *T. cruzi*-specific CD8 T-cell response. Even though these particular mice manage to initially control the levels of parasitemia, they are not able to sustain an appropriate CD8 T-cell response. Therefore, we expect that some of the animals will display an even more deficient *T. cruzi*-specific CD8 T-cell response (number and quality), crucial to maintain control of infection.

Collectively, our results revealed a novel role for Bim in the control of acute phase of *T. cruzi* infection and highlighted the continuous need to investigate molecular mechanisms that control immune responses against pathogens.
