# Correction: Absence of Bim sensitizes mice to experimental *Trypanosoma cruzi* infection

**DOI:** 10.1038/s41419-021-04098-5

**Published:** 2021-08-30

**Authors:** Marcela Hernández-Torres, Rogério Silva do Nascimento, Monica Cardozo Rebouças, Alexandra Cassado, Kely Catarine Matteucci, Maria Regina D’Império-Lima, José Ronnie C. Vasconcelos, Karina R. Bortoluci, José Maria Alvarez, Gustavo P. Amarante-Mendes

**Affiliations:** 1 Instituto de Ciências Biomédicas, Universidade de São Paulo, São Paulo, SP Brazil; 2 Instituto de Investigação em Imunologia, Instituto Nacional de Ciência e Tecnologia (INCT-iii), São Paulo, Brazil; 3 Centro de Terapia Celular e Molecular – CTCMol – Universidade Federal de São Paulo, São Paulo, SP Brazil; 4 Departamento de Farmacologia, Escola Paulista de Medicina, Universidade Federal de São Paulo, São Paulo, Brazil

**Keywords:** Cell death and immune response, Infectious diseases

Correction to: *Cell Death & Disease* 10.1038/s41419-021-03964-6, published online 10 July 2021

The original version of this article unfortunately contained a mistake in the Funding section. The correct funding should read: M.H-T. and K.C.M. were recipients of Ph.D. fellowships from the Fundação de Amparo à Pesquisa do Estado de São Paulo (FAPESP – Brazil - 2012/24737-0, 2015/09568-5). M.C.R. received a postdoctoral fellowship from the Coordenação de Aperfeiçoamento de Pessoal de Nível Superior (CAPES – Brazil). This work was supported by grants from FAPESP (2016/24912-7, 2015/20432-8, 2017/25942-0, 2018/15607-1, 2018/25984-7) and from CNPq (408909/2018-8, 303810/2018-1, 308927/2019-2) to G.P.A-M, M.R.D-L, J.R.V., K.R.B. and J.M.A.

The authors apologize for the error. The original article has been corrected.

